# Serological evidence of vector and parasite exposure in Southern Ghana: the dynamics of malaria transmission intensity

**DOI:** 10.1186/s13071-015-0861-y

**Published:** 2015-04-28

**Authors:** Kingsley Badu, Ben Gyan, Maxwell Appawu, Daniel Mensah, Daniel Dodoo, Guiyun Yan, Chris Drakeley, Guofa Zhou, Ellis Owusu-Dabo, Kwadwo Ansah Koram

**Affiliations:** Department of Immunology, Noguchi Memorial Institute for Medical Research, College of Health Science University of Ghana, LG581, Legon Accra, Ghana; Department of Parasitology, Noguchi Memorial Institute for Medical Research, College of Health Science University of Ghana, LG581, Legon Accra, Ghana; Program in Public Health, Room 3038, Hewitt Hall, College of Health Science, University of California, Irvine, CA 92697-4050 USA; London School of Hygiene and Tropical Medicine, London, UK; Kumasi Centre for Collaborative Research in Tropical Medicine, School of Medical Sciences, Kwame Nkrumah University of Science and Technology, Kumasi, Ghana; Department of Epidemiology, Noguchi Memorial Institute for Medical Research, College of Health Science University of Ghana, LG581, Legon Accra, Ghana

**Keywords:** *Anopheles gambiae*, Salivary peptide, Malaria exposure, Seroprevalence, Seroconversion rate, Merozoite surface protein, Parasite prevalence

## Abstract

**Background:**

Seroepidemiology provides robust estimates for tracking malaria transmission when intensity is low and useful when there is no baseline entomological data. Serological evidence of exposure to malaria vectors and parasite contribute to our understanding of the risk of pathogen transmission, and facilitates implementation of targeted interventions. Ab to *Anopheles gambiae* salivary peptide (gSG6-P1) and merozoite surface protein one (MSP-1_19)_ reflect human exposure to malaria vectors and parasites. This study estimated malaria transmission dynamics using serological evidence of vector and parasite exposure in southern Ghana.

**Methods:**

Total IgG responses to both antigens in an age stratified cohort (<5, 5–14, >14) were measured from South-eastern Ghana. 295 randomly selected sera were analyzed from archived samples belonging to a cohort study that were followed at 3 consecutive survey months (n = 885); February, May and August 2009. Temporal variations in seroprevalence of both antigens as well as differences between the age-stratified cohorts were determined by *χ*^2^ test with p < 0.05 statistically significant. Non-parametric repeated ANOVA – Friedman’s test was used to test differences in antibody levels. Seroprevalence data were fitted to reversible catalytic model to estimate sero-conversion rates.

**Results:**

Whereas parasite prevalence was generally low 2.4%, 2.7% and 2.4% with no apparent trends with season, seroprevalence to both gSG6-P1 and MSP1_19_ were high (59%, 50.9%, 52.2%) and 57.6%, 52.3% and 43.6% in respective order from Feb. to August. Repeated measures ANOVA showed differences in median antibody levels across surveys with specific significant differences between February and May but not August by post hoc Dunn’s multiple comparison tests for gSG6-P1. For MSP1_19_, no differences were observed in antibody levels between February and May but a significant decline was observed from May to August. Seroconversion rates for gSG6-P1 increased by 1.5 folds from February to August and 3 folds for MSP1_19_.

**Conclusion:**

Data suggests exposure to infectious bites may be declining whereas mosquito bites remains high. Sustained malaria control efforts and surveillance are needed to drive malaria further down and to prevent catastrophic rebound. Operational factors for scaling up have been discussed.

## Background

Malaria elimination has again been accepted as the goal of malaria control efforts with thirty-two out of the remaining ninety-nine malaria endemic countries currently implementing malaria elimination strategies [1]. As international efforts towards malaria elimination increase, accurate data on transmission intensity will be crucial for directing control efforts, developing and testing new control tools, and predicting the effects of these interventions under various conditions [2]. It has been suggested that during the pre-elimination phase, the focus of monitoring and evaluation of impact of interventions must shift from surveying health system indicators such as number of malaria cases and associated mortality to measuring malaria transmission intensity and infection [3-5]. However, very low malaria transmission intensity and the non-uniform transmission occurring after periods of extensive control [6,7] highlights important limitations associated with the current tools for measuring malaria transmission intensity. The lack of sensitivity of current malaria transmission tools constitutes a major bottleneck for malaria elimination efforts [3,5].

The entomological inoculation rates (EIR), the product of man biting rates (*Ma*) and the sporozoite rate (*SR,* proportion of mosquitoes carrying sporozoites)*,* is the gold standard for measuring malaria transmission intensity. It is the most direct way of detecting human exposure to infectious bites and mosquito population monitoring. However under conditions of very low malaria transmission the EIR suffers from well recognized limitations [2]. Notably, the intrinsic uncertainty in measuring *Ma* with methods such as human landing catches, resting collections, pyrethrum spray catches, and Centers for Disease Control and Prevention (CDC) light traps are all subject to operator-related variability, such that results may not be reproducible or accurately reflective of the overall local population, and the need for standardized methods for measuring both *Ma* and *SR* [8,9] limit the precision and accuracy of EIR and its potential for measuring a change in transmission. This is especially so at low transmission intensities, where it is difficult to catch sufficient mosquitoes. The limitations associated with measuring malaria transmission by vector mosquitoes are expected to become even more pronounced as ongoing implementation of available control methods, including indoor residual spraying (IRS) and insecticide-treated nets (ITNs), drive down mosquito and malaria endemicity levels [10].

Parasite prevalence (PR), is a well-known metric that is used to estimate the proportion of the human population who are found to be carrying parasites in their blood [11]. The accuracy of outcome varies with the method used [12]. However, it generally becomes less reliable as a tool for measuring the intensity of malaria transmission when parasitemia is low [13]. As a result, more sensitive and standardized metrics are needed to assess transmission intensity in real time, to assess interventions, to acquire data necessary for planning appropriate control programs in areas of low transmission [13,3].

Immuno-epidemiological assays based on human humoral responses to *P. falciparum* and *Anopheles* antigens are potentially valuable for robust transmission measurement [12-15]. In particular, the Merozoite Surface Protein 1 (MSP 1_19_) seroconversion rates has been shown to correlate with malaria transmission intensity (EIR), and to depict malaria endemicity by identifying hotspots of higher malaria transmission [15-18]. MSP-1_19_ seroprevalence and antibody level has proven to be sensitive in discriminating small spatial scales in malaria exposures at varying altitudes, age groups, and distance to *Anopheles* breeding habitats [14,19,20].

The use of antibodies to *Anopheles* salivary proteins as a proxy for human exposure to vector bites and risk of parasite transmission is a promising endeavor. This phenomena rests on the concept that *Anopheles* vectors injects salivary proteins containing a cocktail of bioactive compounds including vasodilators and anticoagulants [21], which mitigate vertebrate host’s defense mechanism such as hemostais, inflammation and thus facilitate blood feeding [22]. Some of the components of the bioactive compounds are antigenic and, elicits adaptive humoral response in the vertebrate host. The level of human exposure to *Anopheles* bites, have thus been found to correlate with the level humoral response to anti-salivary proteins [23,24]. This assay has so far been applied as an epidemiological marker of vector exposure and risk of pathogen transmission in exposed populations. So far, the utility of this application has been demonstrated in leishmaniasis [25], Chagas disease [26] and recently in malaria from western Kenya and elsewhere [20,24-26]. Due to the logistical difficulty in extracting whole saliva from mosquitoes and the possible cross reactivity between common epitopes within the dipteral group the recombinant protein (gSG6) specific to the *Anopheles* genus was isolated and purified for the assay [27-29]. Recently a synthetic peptide, the *gambiae* salivary gland peptide 1 (gSG6-P1) based on the recombinant protein with an enhanced *Anopheles* specificity and antigenecity has been developed and validated [20,30]. The synthetic peptide has standardized the assay and guaranteed high reproducibility such that it is possible to compare results from one lab to the other and from one region to the other. Antibody reactivity to this peptide shows promising characteristics as a biomarker for human biting by *Anopheles* mosquitoes. So far increases in gSG6-P1 specific antibody levels correlated with increased rainfall in a region of very low mosquito exposure and rapid decreases in these levels were observed in individuals after ITNs were introduced in areas of high malaria transmission [31,32]. The gSG6-P1 marker appears to have several characteristics of an ideal biomarker; firstly its very specific to the *Anopheles* genus with no relevant cross-reactivity with epitopes from other proteins or vectors of protozoan parasites [30,32]. Its synthetic nature largely ensures high reproducibility of the assay and it induces specific host humoral response which correlates with the level of exposure to *An. gambiae* bites.

We explored the utility of the *Anopheline* salivary peptide (gSG6-P1) in comparison with MSP-1_19_, a well known malaria antigen, to examine the fine temporal variations in vector and parasite exposure in an area of low malaria transmission but high vector exposure. The ability to detect temporal changes in malaria transmission intensity will enable us to sensibly deploy scarce resources in a targeted focal control to yield maximum community or country benefits and speed malaria elimination.

## Methods

Archived plasma samples for the current study were obtained from an earlier cohort study conducted in Asutuare and its surrounding areas. The cohort was selected from a relatively small geographic area within 5 km radius with non-significant differences in malaria exposure, and malaria antibody genetic markers described in detail by Adu *et al.* [33]. Thus, malaria variation in the cohort, in respect of geographic location was considered homogeneous. Asutuare is a sub-district of Shai Osudoku district, (formerly Dangbe west district) in Greater Accra of Ghana. It is a semi-rural area about 40 km northeast of Accra, the capital of Ghana. The district has a surface area of 1,442 km^2^ and a population size of 122,836 (population and housing census, 2010 (http://www.ghanadistricts.com/pdfs/2010_pop_census_districts.pdf). The population is typically scattered in small satellite villages of about two thousand people. The district usually has two rainy seasons in a year, beginning from April to July and October to December. Malaria transmission is low but perennial, and peaks slightly during and immediately after the major rainy seasons and is lowest during the dry seasons [34]. It is estimated that individuals near the district capital are exposed to about 7.5 infective bites in the rainy season (Personal communication), and 98% of the infections are due to *P. falciparum* [34]. Historically there was a huge sugar processing company with corresponding commercial farms of sugar cane. The community thus has a network of canals created for irrigation schemes. This potentially exposes inhabitants to mosquito bites all year round. It is not surprising that the area has been a site for piloting malaria related interventions since the 1990s [35]. Overall, the sum of 560 participants between the ages of one and thirty were enrolled for the original study in 2009; these were followed up in three cross-sectional community-based surveys to collect blood samples for laboratory analysis; in February towards the end of the dry season, May near the peak of rainy season and August representing snap shots of the perennial transmission in the year. For the purpose of this study, sera from 295 subjects were randomly chosen and assayed from each time point.

### Sample size

Sample size was determined by using the binomial model to estimate the confidence interval (CI) [36]. Because antibody prevalence to the salivary gland protein and the MSP1 were unknown in the area, the antibody prevalence of CSP (25%) previously reported in the cohort [37] was used as the antibody prevalence to estimate the sample size. The sample size with a 95% CI and precision level of 5% was estimated according to the formulae below: In this equation, *n* is the sample size, *z* is the critical value of the standard normal distribution at the 5% level (1.96), *p* is the prevalence of antimalarial CSP antibody, *q* = 1 – *p*, and *d* is the precision level. The population size was estimated to be 2,000. The minimum sample size was estimated to be 288.

$$ {n}_o=\frac{Z_aPq}{d^2} $$

### Ethical approval

The study used sera samples archived from an earlier cohort study conducted in 2009.

Ethical clearance for the main study was granted by the Institutional Review Board (IRB) of the Noguchi Memorial Institute for Medical Research (NMIMR). Study participants or their parents/legal guardians gave written informed consent, which permitted storage for future malaria studies, before enrolment into the study. For the present study, analysis of archived sera was blinded.

### *Anopheles gambiae* salivary antigen

Bioinformatic tools were used to design and optimize the specificity and immunogenicity of the *Anopheles gambiae* salivary peptide (gSG6-P1) as previously reported [30,20]. Protein sequences were sent to Genepep (St-Jean de Vedas, France) which synthesized and purified (>95%) the peptide. This was then shipped to Ghana in lyophilized form.

### Testing for Total IgG antibodies to *An. gambiae* antigen (gSG6-P1)

Serologic testing of human exposure to gSG6-P1 was achieved using Enzyme-Linked Immunosorbent Assay (ELISA) as previously reported [20]. Briefly, gSG6-P1 peptide (20 μg/mL) was used to coat Maxisorp microtitre plates (Roskilde, Denmark) and incubated at 37°C for 2^1^/_2_ hrs and then washed. Blocking buffer (0.5% Casein 0.05% Tween20) was added for 1 hr at room temperature. Sera from participants were diluted at an optimized dilution of 1:20 diluent, and kept at 4°C over night. Polyclonal goat anti-human IgG antibody conjugated to horse radish Peroxidase (Nordic Immunology, Tilburg, Netherlands) at a dilution of 1:10,000 in PBS was used to detect Anti-gSG6- P1 IgG. A peroxidase substrate ABTS (Kirkegaard & Perry Laboratories Inc., Gaithersburg, MD) was then added after washing and kept at room temperature for 50 mins. Enzymatic reaction was stopped with 4 N H_2_SO_4_. Optical density (OD) was then read at 450 nm on a spectrophotometer. Test sera were evaluated in duplicates with a corresponding third well containing no antigen (ODn), a blank well to control for non-specific reactions in the sera and the reagents. IgG levels were determined as final OD computed for each serum as the mean OD value (with antigen) minus the OD value without antigen (ODn-blank well). For the purposes of quality assurance, intra- and inter-assay disparity of control samples was below 20%. Sera whose duplicates had a coefficient of variation (CV) 20% and above were excluded from the analysis. The mean OD of unexposed controls (from the Europe, N = 30) plus 3 SD was used as cut-off value for seropositivity.

### Measurement of anti-human IgG antibodies for *Pf*MSP1_19_ (FVO) antigen

Total IgG antibody to *Pf*MSP1_19_ was measured by indirect ELISA as previously described [14]. The expression and purification of the *Pf*MSP1_19_ recombinant protein has also been described [14,20]. Assay plates (Maxisorp, Roskilde, Denmark) were coated with 0.5 μg *Pf*MSP1 recombinant protein and incubated overnight at 4°C. After blocking, test sera were added in duplicate wells. HRP conjugate of goat antihuman IgG (KPL, Gaithersburg, MD) was added after incubation. Then ABTS (Kirkegaard & Perry Laboratories Inc., Gaithersburg, MD) was added and incubated for 1 h at 22°C. The OD measurements were taken at 414 nm on a spectrophotometer (Spectra- MAX 340PC, Molecular Devices Corporation). Duplicate optical densities were averaged and normalized against a positive control. The cut off for seropositivity was an OD three standard deviations or more above the mean OD obtained in samples from 25 Europeans with no history of previous malaria exposure.

### Data analysis

Seroprevalence was defined as the number of positive responders out of the total number of participants tested. The temporal variations in seroprevalence of both antigens as well as differences between the age-stratified cohorts were determined by *χ*^2^ test with p < 0.05 considered statistically significant. The non-parametric repeated ANOVA – Friedman’s test was used to test if median antibody levels were different across different survey months (Feb, May and August) as well as the different age strata. The Post Hoc Dunn’s multiple comparison tests was used to test specific differences in seroprevalence between specified survey months and age groups. Age specific MSP-1_19_ and gSG6-P1 seroprevalence data was fitted to a simple reversible catalytic model using the maximum likelihood method that assumes a binomial error distribution; *Pt* = *λ / (λ* + *ρ)* [1 – *exp* (− (*λ* + *ρ*) ^t^)] where Pt is the proportion of individuals aged *t* that is seropositive, Lambda *λ* is the annual rate of seroconversion and *ρ* is the annual rate of reversion to seronegative. This was done to investigate the relationship between force of vector and parasite exposure with age. All data were analysed and graphed using GraphPad Prism software (San Diego, CA, USA).

## Results

### Study population and parasite prevalence

A total of 295 subjects had data available for all the 3 surveys (Feb, May and Aug) and thus all analysis were based on the 295 selected samples. 27% out of the subjects were less than 5 yrs whilst the majority of the study subjects were between the ages of 5 and 14. Only 12% of the participants in the selected cohort were in the in the age bracket of 15–29 (Table 1).

**Table 1:** **Total number tested in age stratification, parasite prevalence and antigen specific seroprevalence**

**Age**		***Parasite prevalence (%)***			***χ2***	***P value***
	**N (%)**	**Feb**	**May**	**Aug**		
**<5**	80(27)	2.5	1.3	1.3	0.51	0.77
**5-14**	179(61)	2.8	3.9	3.4	0.34	0.8
**15-29**	36(12)	0.0	0.0	0.0	ND	ND
***χ2***		1.01	2.63	2.05		
***P value***		0.60	0.27	0.36		

### Number of participants, parasite prevalence and antigen specific seroprevalence

Parasite prevalence was generally low with no particular seasonal trends observed across the three contact months (Table 1). Within the age groups, parasite prevalence by microscopy rose from an average of 1.7% in < 5 year group to a two-fold increase (3.4%) in the 5–14 year group but a complete absence (0%) in the adult group. However, Chi square analysis revealed no significant differences in parasite prevalence when the three age groups were compared (Table 1)*.*

### Antigen specific seroprevalence

Exposures to the *Anopheles gambiae* salivary protein (gSG6-P1) were high throughout the three survey months, although a 10% decline in seroprevalence was observed between 1^st^ and 2^nd^ survey months (February and May), this was just at the threshold of statistical significance *(χ2 = 3.6, DF =1, p =0.05).* There was a significant but gradual decline in exposures to the blood stage *P. falciparum* merozoite surface proteins (MSP1_19_.), MSP1_19_ specific seroprevalence observed in 1^st^ survey declined by 5% in the 2^nd^ survey and a further 10% in August (Table 2).

**Table 2:** **Comparison of antigen specific seroprevalence at the population level in different survey months**

***Antigen- specific Seroprevalence***				***χ2***	***s***
	**Feb**	**May**	**August**		
gSG6-P1	59.8	50.9	52.2	4.18	0.123
*Pf*MSP1_19_	57.6	52.3	43.6	6.84	0.033

### Correlation of antigen specific seroprevalence and parasite prevalence with age

**Figure 1:**
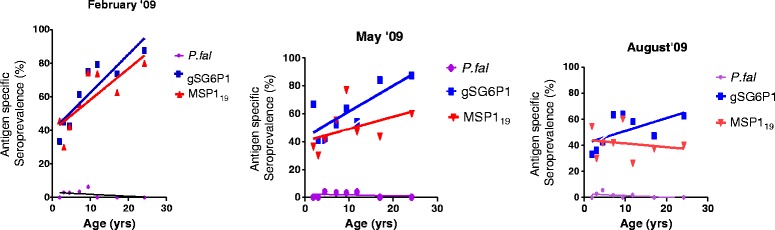
Correlation of antigen specific seroprevalence and parasite prevalence with age.

The spearman correlation analysis revealed an inverse relationship between *P. falciparum* prevalence and age for all three survey months although this was not strong to achieve statistical significance; Feb (r = −0.30; *p* =0.46), May (r = −0.05; *p* =0.93) and August (r = −0.39; *p* =0.32). High exposures to the salivary gland peptide corresponded to strong association with age observed in the first and second survey months (Figure 1), however these exposures gradually declined with a non significant correlation with age in the third survey (r = 0.16; *p* = 0.11). gSG6-P1 responses had very similar trends compared with that observed in MSP1_19_ which also had a strong correlation in the first survey with a gradual decline in the second and third surveys (Figure 1).

### Comparison of antigen-specific median antibody levels at the cohort population level

**Figure 2:**
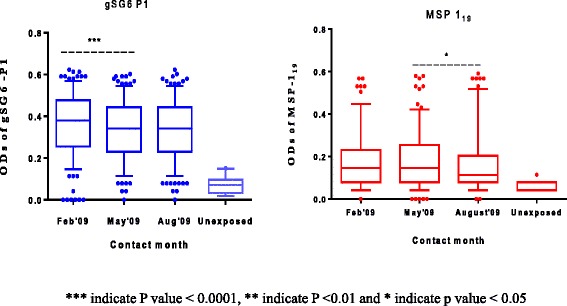
Comparison of median antigen specific antibody levels in within the study cohort.

Median antibody levels of gSG6-P1 and MSP1_19_ antigens as measured in optical densities in the cohort at all survey months across age groups were above the threshold of the unexposed group. Median levels of anti gSG6-P1 antibody showed significant differences in antibody levels in mosquito exposure between the first survey (February 2009) and second (May 2009) survey months, detecting temporal variations in vector exposure among the cohorts at different time points. The Friedman’s test (Repeated measures ANOVA and non parametric) revealed significant differences in antibody levels to gSG6-P1 between the first, second and third survey months at the population level. (F = 33.97; *p* < 0.0001) as well as post hoc Dunn’s multiple comparison tests between Feb and May: (*p* < 0.0001), Feb and August (*p* < 0.0001), but not May and August (*p* > 0.5) (Figure 2).

Similar to the differences in gSG6-P1, the Friedman’s statistic detected overall significant differences in anti MSP1_19_ levels compared across survey months (F = 14.98; *p* = 0.0006). However, post hoc analysis revealed no significant differences between first and second (February and May) survey months (*p* >0.05) but significant decline were observed in the antibody levels between the second and third (May-August) survey months (*p* < 0.05) (Figure 2).

### Age-stratified Cohort and Median antibody levels

**Figure 3:**
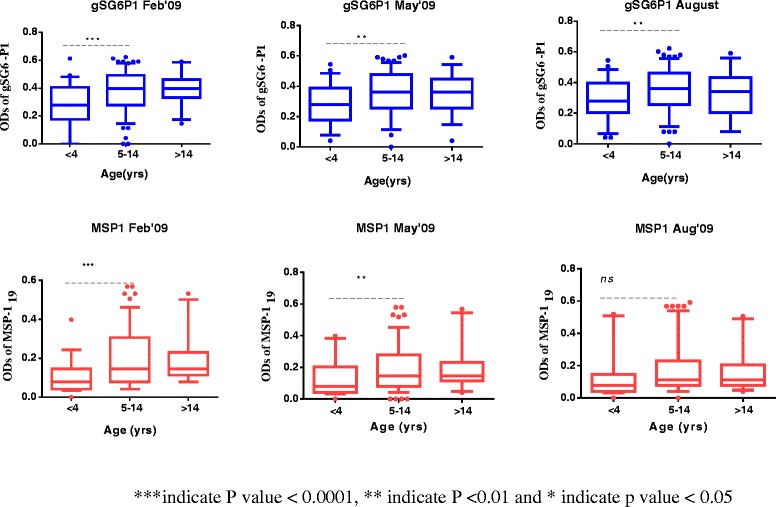
Age-stratified comparisons of antibody levels to specific antigen in contact month.

Generally, median antibody levels to all antigens differed among the age groups throughout the surveys (Kruskal- Wallis H test) (Figure 3). However, post hoc Dunn’s multiple comparisons test revealed differences between specific pairs of age groups.

Kruskal-Wallis H statistic revealed significant differences in anti gSG6-P1 level across all age-stratified cohorts and survey months; Feb (H = 17.49; *p* = 0.0002), May (H =11.09; p = 0.0039) and August (H = 10.43; *p* <0.0054) with increasingly higher antibody levels. However, the Dunn’s multiple comparison tests (post hoc test) between specific pairs of age-stratified cohorts showed intriguing differences: consistently, there were significant differences between < 4 and the 5–14 age groups across all survey months, Feb (*p* < 0.001) May (*p* < 0.01) and Aug (*p* < 0.01) with higher antibody levels observed in the 5–14 year cohorts. However, there were no differences seen in the antibody levels of anti-gSG6P1 in the 5–14 and the >14 age group in the first and second survey months (P > 0.05), a decline in between the third survey with the > 15 year cohort having lower gSG6-P1 levels but this was not statistically significant (Figure 3).

Median antibody levels to the merozoite surface protein (MSP1_19_) increased significantly between <5 and 5–14 age groups in the first and second survey months but not the third (Figure 3). In respective order Feb (H = 20.01; *p* <0.0001) May (H = 9.43; *p* <0.01) August (H = 4.99; *p* > 0.05). Similar to gSG6-P1, there were no differences in the antibody levels observed between the 5–14 and the adult age-groups throughout the three survey months.

### The force of exposure to vector bites and parasite over time

**Figure 4:**
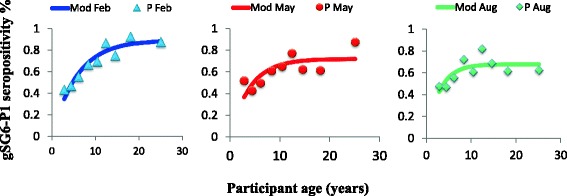
Seroconversion rate gSG6-P1.

**Figure 5:**
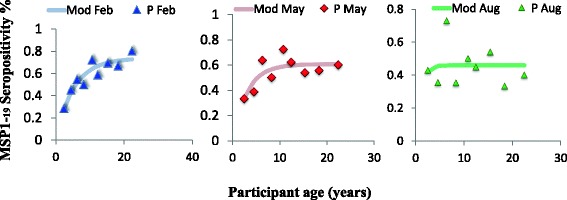
Seroconversion rate MSP1_19_.

The force of exposure to both antigens (gSG6-P1(Figure 4) and MSP1_19_ (Figure 5)) tested increased significantly with age at all contact months, however the rate at which individuals change from being seronegative to seropositive differed with each antigen and survey month, gSG6-P1(Figure 4), MSP1_19_ (Figure 5) . Generally, seroconversion rates (λ) (SCR) for gSG6-P1 and MSP1_19_ gradually increased from February to August, The force of exposure to vector bites increased 1.5 fold from February to August (Figure 4). MSP1_19_ SCR increased marginally between 1.5 folds to 3 folds from February to May then to August. Sero-reversion rates (ρ), also recorded a somewhat corresponding increase (Figure 5).

## Discussion

Metrics of malaria transmission change on different temporal scales, mirroring among other things the changes in vector exposure, parasite infections in humans, as well as the dynamics of changing human immunity. The key component in deciding the appropriateness of malaria transmission metrics as end points for measuring changes in transmission is determined not only by costs, precision, and accuracy, but also by the intrinsic variability of the metric across space and time [38]. Using age specific seroprevalence data, antibody density and seroconversion rates, it is possible to identify temporal variations in mosquito and parasite exposure to show the variation in the intensity of malaria transmission that would otherwise go undetected by parasite prevalence due to apparent low transmission in the Asutuare area.

The use of serological markers of vector and parasite exposure to track changes in malaria transmission over time has several competitive advantages over other metrics. Chiefly, serological markers are more sensitive and robust [15,16,14]. For equivalent parasite rates, serological markers generate seroprevalence that is higher than the parasite prevalence revealing active transmission in progress over a period of time that would otherwise be deemed as interrupted transmission [13,39]. In the current study whereas parasite prevalence was well below 5% throughout the year; equivalent seroprevalence of gSG6-P1 and MSP1_19_ was 40% and above. Similar findings have been reported elsewhere. In Somalia, when no parasites were detected both in wet and dry seasons after screening more than one-thousand people, 17.9% and 19.3% of MSP1_19_ seroprevalence was found respectively [13]. Similarly, in the uphill dwellers of the Western Kenyan highlands it is reported that when parasite prevalence was well below 10%, seroprevalence of gSG6-P1 and MSP1_19_ reached peaks above 50% [20].

Serological markers, in particular gSG6-P1, are robust in tracking temporal changes in vector exposure and risk of pathogen transmission in the younger population under intense transmission intensity [20,27,28,31,32,40]. In the adult population however, there seem to be an immuno-tolerance to the recombinant protein version (gSG6) where higher exposures result in declining immune response, this has been explained as desensitization of the salivary proteins to the immune system of the adults [41]. It is known to be highly antigenic in naïve individuals with only transient exposure and wanes rapidly in the absence of continuous exposure, this has variously been corroborated in Senegal and Angola [31,42,43]. Sagna *et al.* studied human immune response to gSG6-P1 salivary peptide in five communities in northern Senegal where malaria transmission has been described as low, with the *An. gambiae s. l.* as the principal vector. They observed a significant increase in IgG levels to gSG6-P1 during the peak exposure to *Anopheles* bites, and a corresponding decrease right after the end of the exposure season [31]. Moreover, IgG levels to gSG6-P1 varied considerably according to the villages, discriminating the heterogeneity of *Anopheles* exposure between villages [20,31].

In the current study it was observed that Total IgG anti-gSG6-P1 levels showed significant differences between February and May but showed sustained high levels in August. Asutuare is a sugar-cane growing area; a cash crop that thrives in water logged areas, thus the area has several irrigation schemes that virtually flood the area all year round exposing inhabitants to high vector exposure. High vector exposure as evidenced from the anti-gSG6-P1 responses was observed in the month of February, a relatively dry month, this may be due to lagged effect of high rainfall recorded two-three months earlier in 2008 [37]. Meteorological variables have distinct patterns and effects on malaria transmission due to specific lagged correlations and most time series studies have provided evidence of an association between rainfall and mosquito abundance, typically at a single lag of 0, 1 or 2 months depending on the mosquito species [44]. The *Anopheles gambiae,* a principal malaria vector in the district, requires relatively shallow and transient breeding aquatic habitat and therefore its abundance does not peak immediately after heavy rainfall but must tarry for the flooding waters to recede.

MSP1_19_ Age-specific seroprevalence has been used to estimate seroconversion rates (SCR) as a measure of malaria transmission intensity. Earlier studies in Tanzania have shown that these estimates are tightly correlated with EIR measurement [15,16,45]. Age seroprevalence curves reflect different levels of transmission intensity. In low transmission settings development of antibodies is slow and prevalence is higher by the adult population, whereas in a high transmission area, much of the population will be seropositive even at a younger age [18]. The SCR of parasite exposure based on MSP1_19_ seroprevalence has been described as an equivalent of the force of infection and correlated with the entomological inoculation rate. This measures the rate of parasite exposure in the population with age (or time) [15,16]. In the current study the MSP1_19_ SCR increased about 3 fold from February to August. Although seroprevalence decreased approximately 8% (gSG6-P1) and 14% (MSP1_19_) at the population level from the February to August (Table 2), the SCR is a function of age and exposure. It is noteworthy that, when the data was stratified into age cohorts, seroprevalence to both antigens increased significantly from the 1–4 to the 5–14 year groups in all survey months (Figure 3) except for MSP1_19_ in August. Moreover, although parasite prevalence showed a non-significant decline from 2.7% (May) to 2.4% (August), age stratifications revealed that parasite prevalence increased with age in the paediatric population which happens to be 88% of the total study population (Table 1). Parasite prevalence within the >5 year group increased from 2.5%, 1.3% and 1.3% in first (Feb), Second (May) and third (August) survey months to 2.8%, 3.9% and 3.4% respectively. Thus this finding confirms the inherent ability of the SCR to reflect changes in transmission in terms of exposure and time.

The force of exposure to vector bites as seen in seroprevalence and antibody levels in study participants increased 1.5 fold from February to August. This implies that the aggressiveness of vector bites increased or the *Anopheles* population density increased. It has been suggested that the gSG6-P1 is a reliable marker for measuring human–vector contact [32]. Possibly it depicts the human vector contact like the *ma* measured in EIR estimation, this is because human immune response to gSG6-P1 increases and decreases sharply following high and low vector exposure seasons, and transient exposure in naïve individuals, this has been observed by other scientist elsewhere [32,42]. In Lobito, a malaria-endemic coastal city of Western Angola, Papa Drame and coworkers conducted a longitudinal evaluation of the efficient use of insecticide treated net (ITNs) using parasitological, entomological and immunological assessments in children as well as adults in 2010 [32,46]. A significant decrease in anti-gSG6-P1 IgG levels was observed just after the efficient use of ITNs with a subsequent rise in IgG levels to the peptide about four months after when the correct usage of ITN had waned. They thus concluded that the gSG6-P1 “provides a valuable tool in malaria vector control based on a real measurement of human-vector contact” It has also been observed to correspond with malaria endemicity at the population level discriminating villages with higher malaria exposure [27,20,47]. It is thus plausible to state that gSG6-P1 has the inherent ability to track changing vector exposure [32]. It has been shown that gSG6-P1 is able to detect differences in vector exposure in different age groups and distance from *Anopheles* breeding sites [20], rapid buildup and decay of antibody levels [42]. The particular usefulness of gSG6-P1 is its ability to show differences in the short term exposure as has been seen in this study.

Parasite prevalence (PR) in the current study was generally low, with no exposure in the adult population >15 years, this has been reported by other scientists working in the same area [44]. The prevalence was determined solely by microscopy which may have underestimated the prevalence [48]. Parasite prevalence generally tends to be lower in adult population due to their well-developed immunity [49]. However, the strength of the relationship between PR and age depends on the endemicity or the transmission intensity of the area. Similar to this study, no significant relationship was observed between PR and age in the uphill residents of western Kenyan highlands but with low malaria transmission intensities. However, there was a strong inverse relationship in the valley dwellers with relatively higher transmission intensity [14,50]. Under low transmission intensity, specifically when parasite prevalence is below 1–5%, it is complicated to use PR as a metric to detect changes in transmission or evaluate impact of interventions [51]. This has been attributed to several factors associated with the PR. Fundamentally, PR is inherently imprecise because parasite densities fluctuate over the course of a single infection [38], again the accuracy (sensitivity and specificity) of parasite detection in an infected blood depends on the number of parasites/μ of blood with the probability of detecting parasite decreasing at low densities. Both microscopy and RDTs fail to detect subpatent infections when the parasite density is less than 250 parasites per μl [12]. In addition, parasite densities are strongly affected by the recent history of antimalarial drug intake and parasite resistance to those drugs [15]. Thus parasite prevalence is only a discrete measure that represents a snap shot of complex dynamic interactions which may need intensive multiple sampling all year round to be accurate.

Seroprevalence and seroconversion rates of vector (gSG6-P1) and parasite proteins (MSP1_19)_ provide a useful sero-surveillance tool for tracking malaria transmission intensity. In order to scale.

Up this approach, three major issues need to be considered: the source of antigens, collection of study sample and laboratory processing to generate seroprevalence data. The salivary gland antigen is a short sequence synthetic peptide that can be synthesized in-house or by any commercial peptide company. Its synthetic nature generates reproducible results that can be compared across different laboratories [14,30]. Collection of blood in sero-epidemiological surveys used to be very cumbersome especially when antibodies needed to be quantified. However, this has been simplified in recent times, since antibodies can be eluted from filter paper. This has made sample collection and storage uncomplicated [52]. Lastly, the Enzyme Linked-Immuno-sorbent Assay (ELISA) is high throughput, standardizable assay, which is cheap, easy to perform and also generates objective reproducible results. Tusting *et al.* [38], actually quantified the cost per sample with ELISA as USD$0.5 and with a turnover of 1000 samples per week. Other methodologies like the multiplex suspended bead assay (Luminex™) and protein microarrays [53] have the added advantage of analyzing several antigens per sample and require smaller amounts of sera however; these are relatively expensive due to the capital intensive equipments.

## Conclusion

Using antibody levels to gSG6-P1 and MSP1_19_, seroprevalence and seroconversion rates (SCR) together with parasite prevalence, we have identified low parasite prevalence, high vector exposure and small changes in malaria transmission intensity from February through May and then to August 2009. Seroconversion rates for gSG6-P1 increased by 1.5 folds from February to August and 3 folds for MSP1_19_. Possibly, exposure to infectious bites may be declining whereas mosquito bites remains high. Malaria transmission in Asutuare as well as many other areas in Africa where endemicity is low needs careful monitoring with sensitive and robust tools to ascertain progress and to forestall a potential catastrophic rebound.
